# The relationship between LAPTM4B polymorphisms and cancer risk in Chinese Han population: a meta-analysis

**DOI:** 10.1186/s40064-015-0941-7

**Published:** 2015-04-15

**Authors:** Ling-Zi Xia, Zhi-Hua Yin, Yang-Wu Ren, Li Shen, Wei Wu, Xue-Lian Li, Peng Guan, Bao-Sen Zhou

**Affiliations:** China Medical University, Department of Epidemiology, School of Public Health, Shenyang, Peoples R China; University of Liaoning Province, Key Laboratory of Cancer Etiology & Intervent, Shenyang, Peoples R China

**Keywords:** LAPTM4B, Polymorphism, Meta-analysis, Cancer risk

## Abstract

LAPTM4B is a newly cloned gene that shows an active role in many solid tumors progression in substantial researches, mainly through the autophage function. Accumulated studies have been conducted to determine the association of LAPTM4B polymorphism with cancer risk. While the results are inconsistent, we conducted the meta-analysis to determine the strength of the relationship. Results showed that allele*2 carriers exhibited a significantly increased risk of cancer development with comparison to allele*1 homozygote (for *1/2, OR = 1.55, 95% CI 1.367-1.758; for *2/2, OR = 2.093, 95%CI 1.666-2.629; for *1/2 + *2/2, OR = 1.806, 95%CI 1.527-2.137). We also observed a significant association between *2/2 homozygote and cancer risk with comparison to allele*1 containing genotypes (OR = 1.714, 95%CI 1.408-2.088). Allele*2 is a risk factor for cancer risk (OR = 1.487, 95%CI 1.339-1.651). Stratified analysis by tumor type exhibits the significant association of this genetic variants with various cancers. In conclusion, LAPTM4B polymorphism is associated with cancer risk and allele*2 is a risk factor.

**Electronic supplementary material:**

The online version of this article (doi:10.1186/s40064-015-0941-7) contains supplementary material, which is available to authorized users.

## Background

Lysosomes are organelles that contain hydrolytic enzymes such as proteases, nucleases and lipases. The stability of lysosomal membrane and the normality of lysosomal function are essential in cellular biology. Lysosomal-membrane permeabilization (LMP) can pose a threat to cellular homeostasis through release of lysosomal contents, such as lysosomal cathepsins B or D, and is a recognized trigger of cell death (Johansson et al. 2010). Autophagy is a conserved lysosome-mediated intracellular trafficking pathway that degrades and recycles intracellular components, thereby promoting cell survival as well as the adaption to a variety of cell stresses as cancers develop and disseminate (Burman and Ktistakis 2010; Jin and White 2007; Degenhardt et al. 2006; Kenific et al. 2010). Noticeably, too much autophagy may conversely result in autophagic cell death (Scarlatti et al. 2009; Eisenberg-Lerner et al. 2009). Lysosomal retention of drugs is responsible for drug resistance and lysosomal concentration of anthracyclines is considered to increase drug efflux and decrease drug nuclear localization, thereby preventing effective chemotherapy-induced DNA damage (Larsen et al. 2000).

Lysosome-associated protein transmembrane 4 beta (LAPTM4B) is a recently discovered gene mapped to chromosome 8q22.1 with 7 exons and 6 introns spanning at least 50 kb (Shao et al. 2003). LAPTM4B protein has a lysosome localization motif and co-localizes with markers of late endosomes and lysosomes (Shao et al. 2003; Li et al. 2011; Vergarajauregui et al. 2010). LAPTM4B is required for the later stages of autophagy maturation in which autophagosomes are fused with lysosomes to form autolysosomes, the place where cytosol and damaged organelles are discomposed into micro molecules and new macro molecules and ATPs are synthetized, thereby maintaining the cellular homeostasis (Li et al. 2011). The transcriptional products of the gene have been observed over-expressed in many solid tumors (Kasper et al. 2005) and different expression levels exist in tumors of different status (Shao et al. 2003), exerting various functions. Up-expression of LAPTM4B inhibits lysosome-mediated death pathways, promotes autophagy and leads to stress tolerance, thereby enhancing tumor cell growth, survival and resistance to apoptosis. Down regulation of LAPTM4B triggers lysosome membrane permeabilization (LMP), weekening the capability of lysosomes to retain the drug and provoking lysosomal-mediated programmed cell death (Li et al. 2011). Evidences from Maeda, et al. study suggest that the transcriptional product of this gene results in an active role in the progression of cell proliferation other than results from tumor genesis (Maeda et al. 2005) and LAPTM4B proteins play an influential role in occurrence, development, progression and prognosis of cancer cells (Zhou et al. 2008; Yang et al. 2010b; Li et al. 2010b; He et al. 2003; Yang et al. 2010a).

Allelic loss of essential autophage gene can lead to tumor formation in beclin1+/− mutant mice (Qu et al. 2003; Yue et al. 2003; Liang et al. 1999), indicating the role for polymorphisms of autophage-associated gene in tumor suppression. LAPTM4B has two alleles, LAPTM4B allele*1 and LAPTM4B allele*2, encoding proteins with 35kD and 40kD respectively (Liu et al. 2003). Allele*1 contains one copy of a 19-bp sequences at the 5’UTR of the first exon, while the segment is duplicated and tandemly arranged in allele*2. This polymorphism may alter its opening reading frame (ORF) and perform as a cis-acting elememt and participate in genetic translation or tissue specific expression with some transcriptional factors or nucleoproteins (Zhou et al. 2008; Yang et al. 2010b). The relationship between the genetic polymorphisms and cancer susceptibility has been studied while the conclusions are not accordant (Deng et al. 2005; Li et al. 2006; Liu et al. 2007; Sun et al. 2007; Cheng et al. 2008; Sun et al. 2008; Wang and Zhang 2010; Qi 2010; Meng et al. 2011; Fan et al. 2012; Li et al. 2012; Yang et al. 2012; Wang et al. 2012; Zhai et al. 2012; Xu et al. 2012; Meng et al. 2013; Wang et al. 2013; Tang et al. 2014). To evaluate the association of LAPTM4B polymorphisms and the risk of cancer development, we conducted a meta-analysis of 18 enrolled case–control studies including gynecological tumors (Meng et al. 2011; Meng et al. 2013; Xu et al. 2012), digestive system cancers (Wang and Zhang 2010; Zhai et al. 2012; Yang et al. 2012; Qi 2010; Cheng et al. 2008; Liu et al. 2007; Wang et al. 2012; Sun et al. 2008), lung cancer (Li et al. 2006; Deng et al. 2005; Tang et al. 2014), breast cancer (Fan et al. 2012; Li et al. 2012), nasopharyngeal carcinoma (Wang et al. 2013), and lymphoma (Sun et al. 2007).

## Results

### Characteristics of the studies

**Figure 1:**
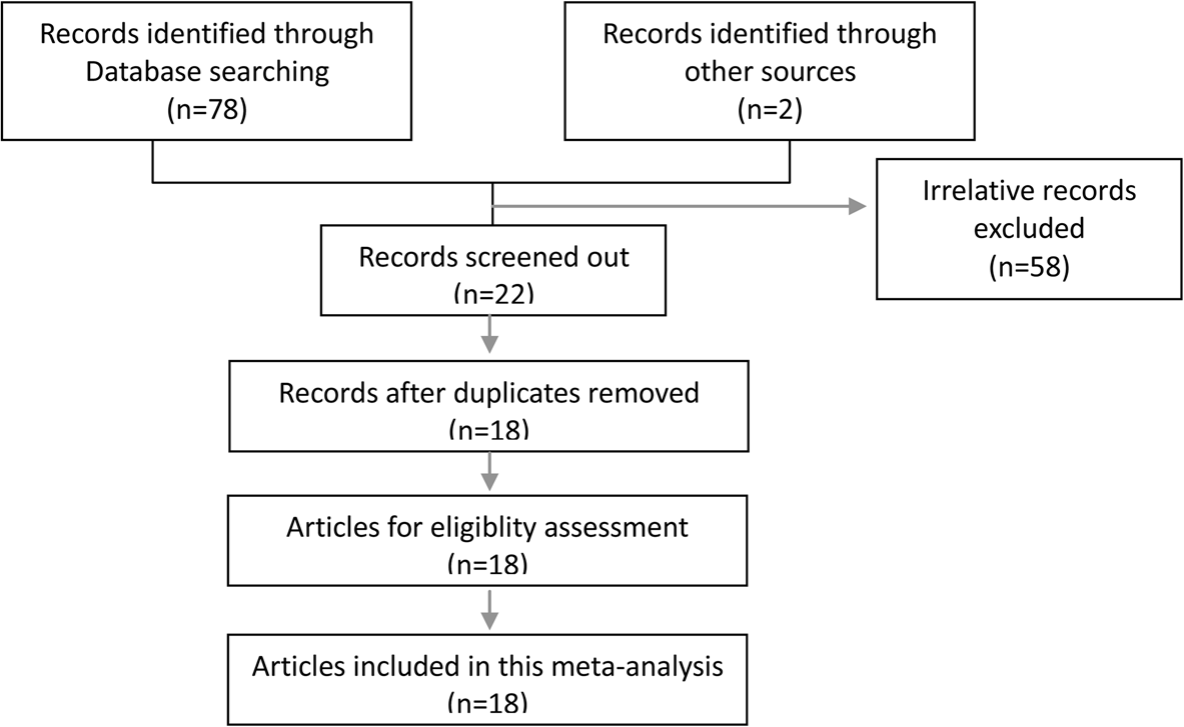
The flowchart of the selection process.

The flowchart of the selection of eligible studies is shown as Figure 1. The major information of the publications is listed in Table 1.Totally, 18 eligible case–control studies published from 2005 to 2014 were recruited in this meta-analysis. The countries of origin and ethnic group of these studies are not shown in the table for the study populations are all Chinese. All of the enrolled studies comply with Hardy-Weinberg Law.

**Table 1:** **Characteristics of studies eligible in the meta-analysis**

**Author**	**Year**	**Type of tumor**	**Numbers**		**Genotype(case/control)**			**MAF (%)**	**P(HWE)**
			**case**	**control**	*1/1	*1/2	*2/2		
Deng et al. (2005)	2005	Lung cancer	166	134	54/67	91/59	21/8	75(28.0)	*0.2850*
Li et al. (2006)	2006	Lung cancer	131	104	70/57	56/36	5/11	58(27.9)	*0.1554*
Liu et al. (2007)	2007	Gastric cancer	214	350	88/199	107/133	19/18	169(24.1)	*0.4835*
Sun et al. (2007)	2007	lymphoma	166	350	72/199	71/133	23/18	169(24.1)	*0.4835*
Cheng et al. (2008)	2008	Colon cancer	253	350	113/199	112/133	28/18	169(24.1)	*0.4835*
		Rectal cancer	237	350	126/199	101/133	10/18	169(24.1)	*0.4835*
		esophageal cancer	211	350	123/199	80/133	8/18	169(24.1)	*0.4835*
Sun et al. (2008)	2008	hepatocellular	190	175	72/99	110/67	8/9	85(24.3)	*0.587*
Wang and Zhang (2010)	2010	Pancreatic cancer	58	156	24/74	26/67	8/15	97(31.1)	*0.9766*
Qi (2010)	2010	liver	86	77	27/36	51/34	8/7	48(31.2)	*0.7985*
Meng et al. (2011)	2011	Cervical cancer	317	416	127/225	153/163	37/28	219(26.3)	*0.8352*
Fan et al. (2012)	2012	breast	732	649	326/346	342/262	64/41	344(26.5)	*0.3556*
Li et al. (2012)	2012	breast	208	211	90/129	100/76	18/6	88(20.9)	*0.1853*
Yang et al. (2012)	2012	Gallbladder cancer	91	155	34/88	45/57	12/10	77(24.8)	*0.8508*
Wang et al. (2012)	2012	liver	303	515	107/272	156/205	40/38	281(27.3)	*0.9415*
Zhai et al. (2012)	2012	hepatocellular	102	135	37/	52/	13/	(24.07)	*-*
Xu et al. (2012)	2012	ovarian	282	365	122/231	115/108	45/26	160(21.9)	*0.64*
Meng et al. (2013)	2013	Endometrial cancer	283	378	93/200	135/140	55/38	216(28.6)	*0.0718*
Wang et al. (2013)	2013	nasopharyngeal cancer	134	327	74/163	48/145	12/19	183(28.0)	*0.0700*
Tang et al. (2014)	2014	NSCLC	392	437	158/226	171/176	63/35	246(28.1)	*0.9284*

*MAF, minor allele frequency, in this study, minor allele refers to allele*2, P (HWE), P value for test of Hardy-Weinberg equilibrium.

### Main results

**Figure 2:**
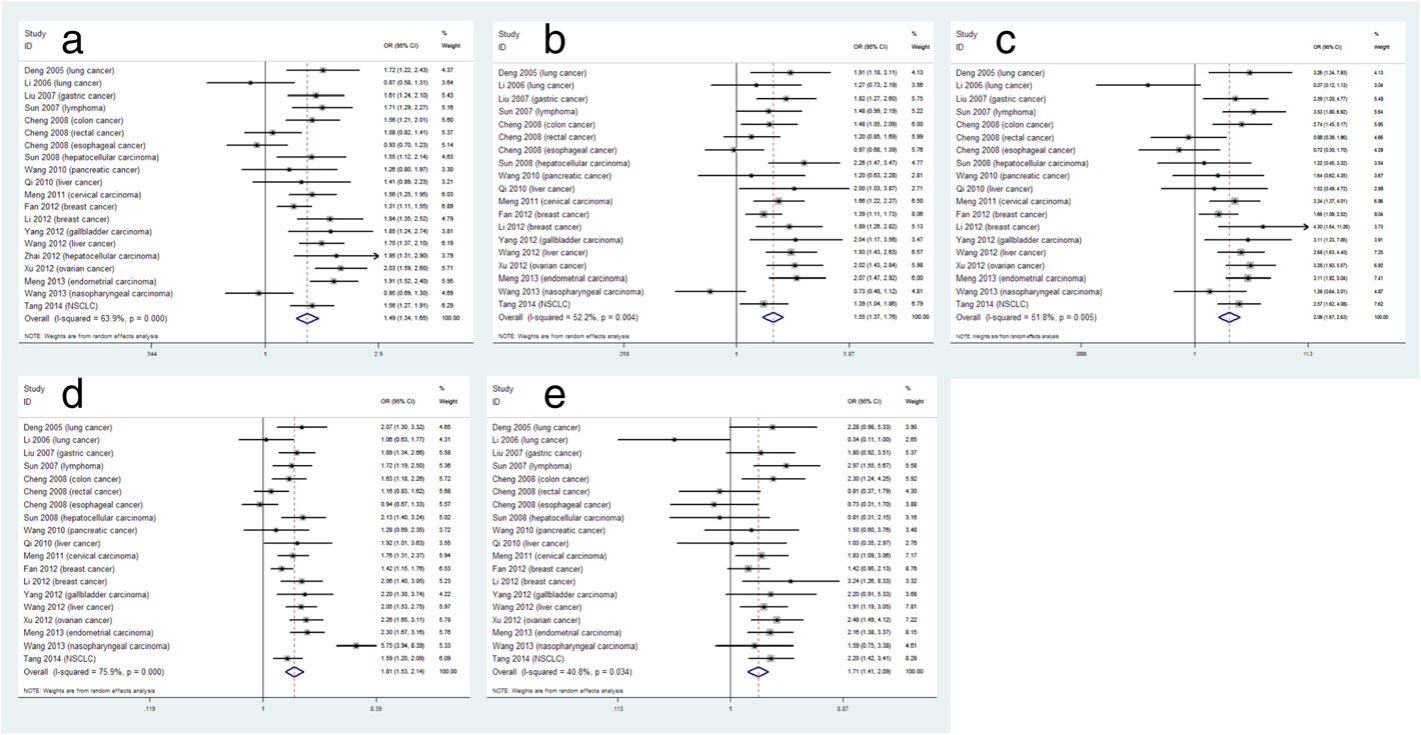
The pooled Ors and 95%CIs for the relationship between LAPTM4B polymorphism and cancer susceptibility. The forest plot for relationship between LAPTM4B polymorphism and cancer susceptibility for *2 vs *1 **(a)**, *1/2 vs *1/1 **(b)**, *2/2 vs *1/1 **(c)**, *1/2 + *2/2 vs *1/1 **(d)**, *2/2 vs *1/2 + *1/1 **(e)**.

Table 2 shows the main results of pooled OR and 95% CI for LAPTM4B polymorphism and cancer risk. Generally speaking, allele*2 carriers showed a significant association with cancer risk. When adjusted by age, gender, et al., the association between LAPTM4B polymorphism and cancer susceptibility is also observed (Additional file 1: Table S1). The forest plots of the pooled OR and 95% CI for the five genetic models are shown as Figure 2.

**Table 2:** **The pooled ORs and 95%CIs for the association between LAPTM4B polymorphism and cancer susceptibility**

**Models**	**No. of studies**	**No. of population (case/control)**	**OR**	**95%CI**	**P**	**P(H)**	**P(P)**
*2 vs *1	18	4556/4584	1.487	1.339-1.651	<0.001	<0.001	0.667
*1/2 vs *1/1	17	4454/4449	1.550	1.367-1.758	<0.001	0.004	0.759
*2/2 vs *1/1	17	4454/4449	2.093	1.666-2.629	<0.001	0.005	0.099
*1/2 + *2/2 vs *1/1	17	4454/4449	1.806	1.527-2.137	<0.001	<0.001	0.564
*2/2 vs *1/2 + *1/1	17	4454/4449	1.714	1.408-2.088	<0.001	0.034	0.064

*P(H): P-value for test of heterogeneity, P(P): P value for test of publication bias.

Another meta-analysis for the estimation of association between LAPTM4B expression and cancer prognosis was conducted. Results are shown in Additional file 2: Table S3. Overexpression of LATPM4B may be a risk predictor for poor survival. The articles eligible for this meta-analysis are listed in Additional file 3: Table S2.

### Subgroup analysis results

The results of stratified analysis by tumor type are listed in Table 3. Stratified analysis results suggested that allele*2 may increase the risk of multiple cancers. Overexpression of LAPTM4B is also associated with poor prognosis in multiple cancers (Additional file 4: Table S4).

**Table 3:** **Stratified analysis for the association between LAPTM4B polymorphism and cancer risk by tumor type**

**Type of tumor**	**Num**	*2 vs *1		*1/2 vs *1/1		*2/2 vs *1/1		*1/2 + *2/2 vs *1/1		*2/2 vs *1/2 + *1/1	
		**OR(95%CI)**	**P(H)**	**OR(95%CI)**	**P(H)**	**OR(95%CI)**	**P(H)**	**OR(95%CI)**	**P(H)**	**OR(95%CI)**	**P(H)**
Digestive system	8	1.444(1.313-1.588)**	0.011	1.557(1.365-1.777)**	0.034	1.873(1.460-2.402)**	0.082	1.594(1.404-1.809)**	0.009	1.499(1.179-1.906)**	0.204
Gastrointestinal	2	1.334(1.176-1.513)**	0.003	1.332(1.119-1.584)**	0.084	1.603(1.125-2.285)*	0.024	1.359(1.149-1.607)**	0.020	1.400(0.990-1.981)	0.069
Gland	6	1.603(1.387-1.853)**	0.687	1.929(1.573-2.364)**	0.615	2.186(1.539-3.104)**	0.524	1.976(1.625-2.403)**	0.677	1.597(1.144-2.228)*	0.462
Gynecological tumor	3	1.810(1.583-2.069)**	0.253	1.891(1.563-2.287)**	0.584	2.900(2.156-3.901)**	0.638	2.073(1.733-2.480)**	0.400	2.144(1.619-2.839)**	0.716
Breast cancer	2	1.413(1.222-1.635)**	0.058	1.488(1.227-1.806)**	0.187	1.959(1.338-2.868)**	0.075	1.549(1.286-1.865)**	0.101	1.640(1.132-2.376)*	0.116
Lung cancer	3	1.454(1.236-1.710)**	0.026	1.467(1.169-1.842)**	0.458	2.073(1.431-3.002)**	0.004	1.563(1.260-1.939)**	0.164	1.748(1.226-2.492)*	0.006
Others	2	1.309(1.060-1.615)*	0.007	1.063(0.797-1.416)	0.017	2.337(1.420-3.847)**	0.075	2.860(2.184-3.745)**	<0.001	2.278(1.403-3.698)**	0.220

*P < 0.05, **P ≤ 0.001, Num: the number of studies, P (H): the P value for test of heterogeneity among studies, Digestive system; include references (Liu et al. 2007; Cheng et al. 2008; Sun et al. 2008; Wang and Zhang 2010; Qi 2010; Yang et al. 2012; Wang et al. 2012; Zhai et al. 2012), Gastrointestinal: include references (Liu et al. 2007; Cheng et al. 2008), Gland: include references (Sun et al. 2008; Wang and Zhang 2010; Qi 2010; Yang et al. 2012; Wang et al. 2012; Zhai et al. 2012), Gynecological tumor: include references (Meng et al. 2011; Xu et al. 2012; Meng et al. 2013), Breast cancer: include references (Fan et al. 2012; Li et al. 2012), Lung cancer: include references (Deng et al. 2005; Li et al. 2006; Tang et al. 2014), others: includes Lymphoma (Sun et al. 2007) and Nasopharyngeal carcinoma (Wang et al. 2013).

### Sensitivity analysis

A sensitivity analysis was performed by omitting every study in turn to find potential outliers to check the inclusion criteria. Pooled estimates for all genetic models were insensitive to the removal of individual study, and the corresponding pooled ORs were not substantially altered, indicating that our results were stable and reliable (data not shown).

### Publication bias

**Figure 3:**
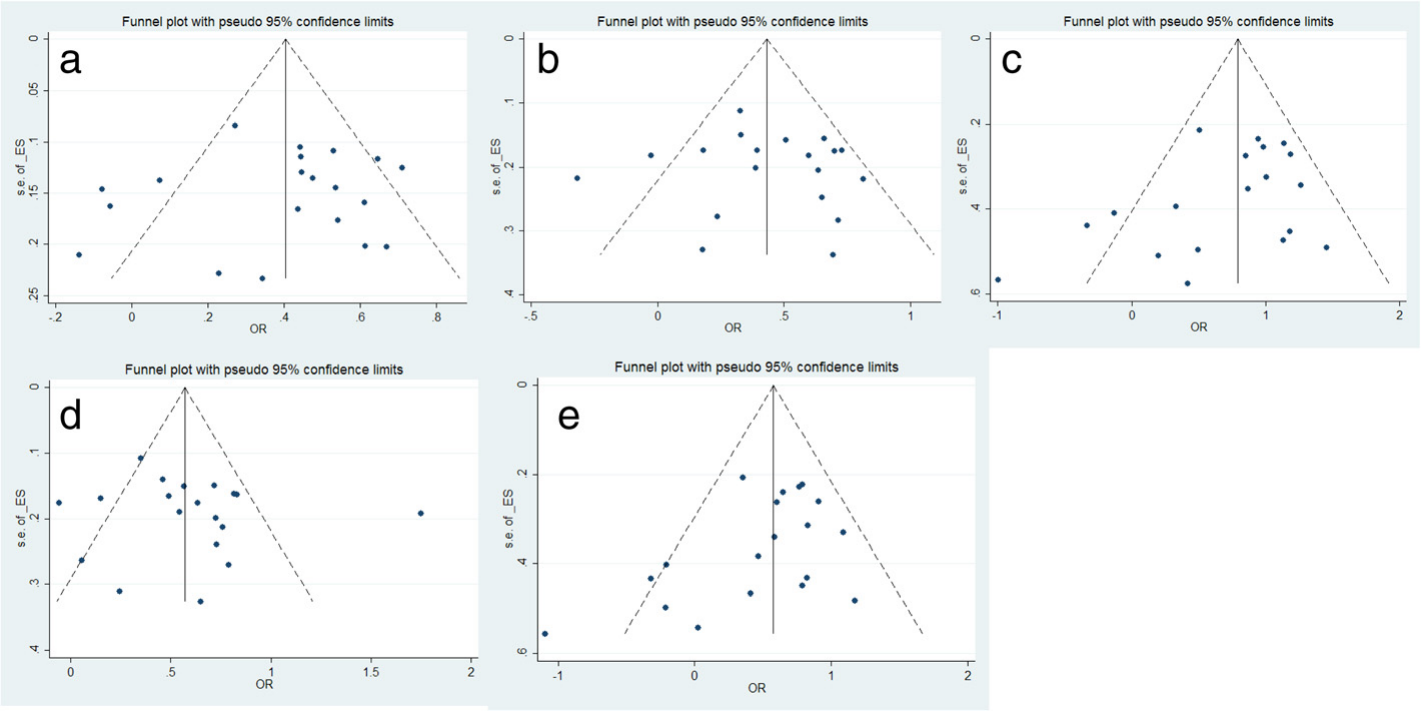
The funnel plot for the test of publication bias. The funnel plot for *2 vs *1 **(a)**, *1/2 vs *1/1 **(b)**, *2/2 vs *1/1 **(c)**, *1/2 + *2/2 vs *1/1 **(d)**, *2/2 vs *1/2 + *1/1 **(e)**.

Begger’s plot funnel and Egger’s test were performed to examine the underlying publication bias. The symmetrical plot funnel and p value >0.05 for Egger’s test indicated no publication bias in this meta-analysis (Figure 3).

## Discussion

Due to insufficient angiogenesis, tumors frequently experience elevated metabolic stress from nutrient and oxygen deprivation (Jin and White 2007; Folkman 2003). Survival mechanisms such as autophagy may be more reliable for cancer cells to maintain growth or to disseminate (White and DiPaola 2009). Many studies have shown the roles of LAPTM4B in limiting cell death and promoting autophagy (Li et al. 2011; He et al. 2003; Yang et al. 2010a).

To date, the exact molecular mechanism that might be involved in progressive phenotypes caused by LAPTM4B protein remains unclear. Nonetheless, accumulated detections in other studies may demonstrate the functional mechanism to some extent.

Both the C- and N-terminal of LAPTM4B protein contain a proline-rich domain PXXP, forming a specific src-homology 2 (SH2) binding domain at C-terminal and specific src-homology 3(SH3) binding domain at N-terminal. The functional domain in C-terminal has a lysosome localization motif, sharing the same domain with the markers of endosomes and lysosomes (Liu et al. 2004; Hunziker and Geuze 1996; Honing et al. 1998; Dell’Angelica et al. 2000; Hogue et al. 2002). And the N-terminal offers sites for SH3-containing proteins such as phosphoinositide 3-kinase (PI3K), protein phosphatase 2A and protein kinase C (PKC) (Shao et al. 2003; Liu et al. 2004). We hypothesized that C-terminal is responsible for positioning and N-terminal is responsible for the function. LAPTM4B plays a significant role in cell cycle progression through regulation of c-Myc, cyclinD1/E and p27 (He et al. 2003; Yang et al. 2010a; Liu et al. 2009). Up-expression of LAPTM4B proteins promoted the phosphorylation of AKT, the pivotal signaling pathway in cell survival and proliferation (Cantley 2002; Yang et al. 2010a; Medema et al. 2000). Moreover, LAPTM4B can decrease the expression of p16, increase the expression of c-Fos, c-Jun (Yang et al. 2010a; Liu et al. 2009; Zhou et al. 2010b), and phosphorylate Rb (Retinoblastoma protein) (Yang et al. 2010a). Another study (Li et al. 2010a) suggested that LAPTM4B may promote drug efflux and multidrug resistance through PI3K/AKT pathway and thus results in poor prognosis.

A high level of expression of LAPTM4B protein has been observed in cells with tendency of migration and metastasis, while the knockdown of the genetic expression reverses the propensity of growth, proliferation and malignancy (Li et al. 2010b; He et al. 2003; Liu et al. 2009). In Zhou, et al. (Zhou et al. 2010a) study, we observed that the over-expression of LAPTM4B enhanced the expression of MMP-2, MMP-9 and uPA (Milde-Langosch 2005). Among them, MMP-9 and uPA are targets of activator protein (AP-1) (Milde-Langosch 2005). Given the fact that no binding site of AP-1 was found in promoter region of MMP-2 gene (Yan and Boyd 2007) and that uPA can activate MMP-2 (Mazzieri et al. 1997), we inferred that LAPTM4B might activate MMP-2 by up-regulating expression of uPA.

The allele LAPTM4B*1differs from allele LAPTM4B*2 in that it contains a particular single 19-bp sequence, whereas LAPTM4B *2 contains two copies of these sequences in the 5’UTR of exon 1. This results in the change in the N terminal of the proteins. Li (Li et al. 2012) and Wang (Wang et al. 2012) also observed that the allele LAPTM4B*2 is associated with higher level of LAPTM4B expression.

With the detections mentioned above, we hypothesized that the effect of the genetic polymorphism in LAPTM4B on cancer susceptibility may result from the change in N-terminal region or from the change in expression level with different genotypes.

While the results of the studies concerning the relationship between LAPTM4B polymorphism and cancer risk are inconsistent, we conducted this meta-analysis to determine the strength of the association with a relatively large sample size containing 4556 cases and 4584 controls. In our meta-analysis, the association between LATPM4B polymorphism and cancer susceptibility and the association between LAPTM4B expression levels and cancer prognosis were observed in multiple cancers. All above support our hypotheses of the association between LAPTM4B polymorphism and cancer susceptibility.

This analysis has some limitations. One of the limitations lies in the study population. All the populations are Chinese. This may because that the gene is newly discovered and the polymorphisms were initially detected in Chinese Han population. And that our analysis indicated a strong relationship between this polymorphism and cancer risk, this meta-analysis may be a reference for further researches in other countries. Second, the lack of raw data, including the information of histology, family history and smoking status, limited the assessment of interaction between gene-gene and gene-environment.

Asides the limitations mentioned above, this study presents some advantages. No publication bias and heterogeneity among studies were observed in this analysis, suggesting a stable and reliable result. Additionally, the estimation of pooled ORs and 95%CIs was based on a relatively large scale of cases and controls. In this view, the results indicated a powerful association between the genetic polymorphisms and cancer risk and may provide a strong evidence for further researches.

## Conclusions

In summary, LAPTAM4B polymorphisms are associated with multiple cancer susceptibility and allele*2 is a risk factor for various tumors. More studies on the association of LAPTM4B polymorphisms and cancer risk in other countries are warranted to confirm the association.

## Methods

### Identification and eligibility of relevant study

All of the studies were identified through five online electronic databases including PubMed, web of science, google scholar, CNKI and WanFang database, using the search strategies “keywords or subjects or title or abstract = (LAPTM4B AND polymorphism AND (cancer OR carcinoma OR tumor OR sarcoma))”. Citation indexes were performed and the reference list was searched to identify potential relevant studies. The last search update was Jun 29, 2014.

Studies involved in the meta-analysis should meet the following inclusion criteria: (i) a case–control study; (ii) frequency of genotype or allele is available; (iii) P value of Hardy-Weinberg equilibrium for the distribution of genotypes in control group should be no less than 0.05.

Articles will be excluded for: (1) no available data to calculate OR or no original data to estimate the P value of Hardy-Weinberg equilibrium; (2) duplicate publication; (3) meeting records or articles written neither in English nor in Chinese.

### Data extraction

Two authors worked independently to extract information carefully. Any disagreement would be discussed until a consensus was reached between two authors. Otherwise, another author would check up the controversial information and a final conclusion would be drawn from the vote results. The information collected from each study contained: the surname of first author, the publication year, country of origin, ethnic group, type of tumor, frequency of genotypes and alleles in cases and controls.

### Statistic analysis

The strength of the association of LAPTM4B polymorphism and cancer risk is measured by odd ratios (ORs) with 95% confidence intervals (CIs). The statistical significance of the pooled ORs is determined by Z-test. We estimated the cancer risk related to allele*2, compared to allele*1. For genotypes, we estimated the OR associated with genotype *1/2, *2/2 and allele*2 containing genotypes (*1/2 + *2/2), compared with *1/1 homozygote, respectively. Stratified analysis was performed according to the type of tumor.

Hardy-Weinberg equilibrium (HWE) was tested by chi-square goodness of fit and a P value no less than 0.05 indicates that the study complies with Hardy-Weinberg Law. The degree of heterogeneity among studies was investigated by Q-statistic. A P value >0.05 indicated a lack of heterogeneity among studies and we chose the fixed effect model (the Mantel-Haenszel method). Otherwise, we selected a random effect model (DerSimonian–Laird method). A funnel plot was used to decide the presence of publication bias, in which the standard error of log (OR) of each study was plotted against its log (OR) and an asymmetric plot suggested the existence of publication bias. Egger’s test was then performed to verify the presence and determine the degree of asymmetry. P < 0.05 was considered to indicate the statistically significant publication bias. A sensitivity analysis was carried out to identify the potential outliers. P values were all based on two-way tests. The meta-analysis was performed in STATA version 11.0.

## Additional files

Additional file 1: Table S1. The pooled adjusted OR and 95%CI for the estimation of association between LAPTM4B polymorphism and cancer susceptibility. Additional file 2: Table S3. The pooled HR and 95%CI for the estimation of association between LAPTM4B expression and cancer prognosis. Additional file 3: Table S2. The eligible articles included in this meta-analysis for the estimation of association between LAPTM4B expression and cancer susceptibility. Additional file 4: Table S4. The stratified analysis for the estimation of association between LAPTM4B expression and prognosis in different type of cancer.

## Abbreviations

LAPTM4B: Lysosome-associated protein transmembrane 4 beta
OR: Odds ratio
CI: confidence interval
LMP: Lysosomal-membrane permeabilization
SH2: src-homology 2
SH3: src-homology 3
PI3K: Phosphoinositide 3-kinase
PKC: Protein kinase C
