# Supplementary material for: The relationship between LAPTM4B polymorphisms and cancer risk in Chinese Han population: a meta-analysis
Source: Springerplus. 2015 Apr 15;4:179. doi: 10.1186/s40064-015-0941-7 (PMC4408309; doi:10.1186/s40064-015-0941-7)
Supplement: Additional file 1: Table S1. — The pooled adjusted OR and 95%CI for the estimation of association between LAPTM4B polymorphism and cancer susceptibility. [file 40064_2015_941_MOESM1_ESM.docx]

| Models | No. of studies | OR | 95%CI | P |
| --- | --- | --- | --- | --- |
| *2 vs *1 | 10 | 1.782 | 1.570-2.028 | <0.001 |
| *1/2 vs *1/1 | 10 | 2.065 | 1.675-2.547 | <0.001 |
| *2/2 vs *1/1 | 10 | 1.791 | 1.483-2.163 | <0.001 |
| *1/2 + *2/2 vs *1/1 | 6 | 2.500 | 1.941-2.331 | <0.001 |

Table S1 The pooled adjusted OR and 95%CI for the estimation of association between LAPTM4B polymorphism and cancer susceptibility
