# Supplementary material for: The relationship between LAPTM4B polymorphisms and cancer risk in Chinese Han population: a meta-analysis
Source: Springerplus. 2015 Apr 15;4:179. doi: 10.1186/s40064-015-0941-7 (PMC4408309; doi:10.1186/s40064-015-0941-7)
Supplement: Additional file 2: Table S3. — The pooled HR and 95%CI for the estimation of association between LAPTM4B expression and cancer prognosis. [file 40064_2015_941_MOESM2_ESM.docx]

Table S3 The pooled HR and 95%CI for the estimation of association between LAPTM4B expression and cancer prognosis.

| Model | No. of studies | No. of population | HR | 95%CI | P | P(H) |
| --- | --- | --- | --- | --- | --- | --- |
| OS | 9 | 977 | 3.931 | 2.897-5.332 | <0.001 | 0.066 |
| PFS | 3 | 300 | 4.378 | 1.460-13.129 | 0.008 | 0.003 |
| DFS | 5 | 579 | 3.644 | 2.393-5.547 | <0.001 | 0.172 |

OS: overall survival, PFS: progression-free survival, DFS: disease-free survival, P(H):p-value for the test of heterogeneity
