# Supplementary material for: The relationship between LAPTM4B polymorphisms and cancer risk in Chinese Han population: a meta-analysis
Source: Springerplus. 2015 Apr 15;4:179. doi: 10.1186/s40064-015-0941-7 (PMC4408309; doi:10.1186/s40064-015-0941-7)
Supplement: Additional file 3: Table S2. — The eligible articles included in this meta-analysis for the estimation of association between LAPTM4B expression and cancer susceptibility. [file 40064_2015_941_MOESM3_ESM.docx]

Table S2 The eligible articles included in this meta-analysis for the estimation of association between LAPTM4B expression and cancer susceptibility.

| author | year | Type of tumor | No. | estimates | HR | 95%CI |
| --- | --- | --- | --- | --- | --- | --- |
| Zhou L([Zhou et al. 2007](#_ENREF_12)) | 2007 | GBC | 75 | OS(score) | 2.853 | 1.391-5.850 |
|  |  |  |  | DFS(score) | 2.443 | 1.104-5.405 |
| Zhou L([Zhou et al. 2008](#_ENREF_13)) | 2008 | extrahepatic cholangiocarcinoma | 81 | OS(score) | 2.411 | 1.082-5.373 |
|  |  |  |  | DFS(score) | 2.016 | 1.124-3.617 |
| Yang Y([Yang et al. 2008](#_ENREF_7)) | 2008 | Ovarian carcinoma | 85 | OS | 3.37 | 1.33-8.52 |
|  |  |  |  | PFS | 2.33 | 1.07-5.09 |
| Yang H([Yang et al. 2010a](#_ENREF_5)) | 2010 | HCC | 65 | OS | 3.226 | 1.348-7.718 |
|  |  |  |  | DFS | 2.082 | 1.025-4.229 |
| Meng F([Meng et al. 2010](#_ENREF_2)) | 2010 | Cerical carcinoma | 113 | OS | 4.357 | 1.326-14.320 |
|  |  |  |  | DFS | 4.297 | 1.306-14.139 |
| Yang H([Yang et al. 2010b](#_ENREF_6)) | 2010 | HCC | 71 | OS | 3.123 | 1.221-7.992 |
|  |  |  |  | DFS | 4.418 | 1.856-10.518 |
| Yin M([Yin et al. 2011](#_ENREF_8)) | 2011 | Ovarian carcinoma | 113 | OS | 20.611 | 5.916-71.808 |
|  |  |  |  | PFS | 17.852 | 6.31-50.51 |
| Kang Y([Kang et al. 2012](#_ENREF_1)) | 2012 | CRC | 136 | OS | 22.774 | 5.287-98.091 |
|  |  |  |  | DFS | 11.674 | 3.562-38.263 |
| Yin M([Yin et al. 2012](#_ENREF_9)) | 2012 | Ovarian carcinoma | 102 | OS | 2.644 | 1.439-4.858 |
|  |  |  |  | PFS | 2.489 | 1.388-4.461 |
| Zhang G([Zhang et al. 2012](#_ENREF_10)) | 2012 | Pancreatic carcinoma | 98 | OS | 3.484 | 1.764-6.897 |
| Xiao M([Xiao et al. 2013](#_ENREF_4)) | 2013 | Brest cancer | 194 | OS | 4.546 | 1.067-19.364 |
|  |  |  |  | DFS | 3.327 | 1.176-9.408 |
| Tang H([Tang et al. 2014](#_ENREF_3)) | 2014 | NSCLC | 186 | OS | 1.567 | 1.028-2.389 |
| Zhang H([Zhang et al. 2014](#_ENREF_11)) | 2014 | Prostate cancer | 180 | RFS | 1.848 | 1.268-2.692 |

*No: the number of population, GBC:gallbladder carcinoma, HCC:hepatocellular carcinoma, CRC:colorectal cancer, NSCLC: non-small cell lung cancer, OS: overall survival, DFS: disease-free survival, RFS: recurrence-free survival, PFS: progression-free survival.

References:

Kang Y, Yin M, Jiang W, Zhang H, Xia B, Xue Y, Huang Y (2012) Overexpression of LAPTM4B-35 is associated with poor prognosis in colorectal carcinoma. American journal of surgery 204 (5):677-683. doi:10.1016/j.amjsurg.2012.02.003

Meng F, Luo C, Hu Y, Yin M, Lin M, Lou G, Zhou R (2010) Overexpression of LAPTM4B-35 in cervical carcinoma: a clinicopathologic study. International journal of gynecological pathology : official journal of the International Society of Gynecological Pathologists 29 (6):587-593. doi:10.1097/PGP.0b013e3181e0898e

Tang H, Tian H, Yue W, Li L, Li S, Gao C, Si L, Qi L, Lu M (2014) Overexpression of LAPTM4B is correlated with tumor angiogenesis and poor prognosis in non-small cell lung cancer. Medical oncology 31 (6):974. doi:10.1007/s12032-014-0974-8

Xiao M, Jia S, Wang H, Wang J, Huang Y, Li Z (2013) Overexpression of LAPTM4B: an independent prognostic marker in breast cancer. Journal of cancer research and clinical oncology 139 (4):661-667. doi:10.1007/s00432-012-1368-y

Yang H, Xiong F, Qi R, Liu Z, Lin M, Rui J, Su J, Zhou R (2010a) LAPTM4B-35 is a novel prognostic factor of hepatocellular carcinoma. Journal of surgical oncology 101 (5):363-369. doi:10.1002/jso.21489

Yang H, Xiong FX, Lin M, Yang Y, Nie X, Zhou RL (2010b) LAPTM4B-35 overexpression is a risk factor for tumor recurrence and poor prognosis in hepatocellular carcinoma. Journal of cancer research and clinical oncology 136 (2):275-281. doi:10.1007/s00432-009-0659-4

Yang Y, Yang H, McNutt MA, Xiong F, Nie X, Li L, Zhou R (2008) LAPTM4B overexpression is an independent prognostic marker in ovarian carcinoma. Oncology reports 20:1077-1083. doi:10.3892/or_00000112

Yin M, Li C, Li X, Lou G, Miao B, Liu X, Meng F, Zhang H, Chen X, Sun M, Ling Q, Zhou R (2011) Over-expression of LAPTM4B is associated with poor prognosis and chemotherapy resistance in stages III and IV epithelial ovarian cancer. Journal of surgical oncology 104 (1):29-36. doi:10.1002/jso.21912

Yin M, Lou C, Zhang W, Meng F, Zhang H, Ning X, Zhou R, Dong X, Lou G (2012) LAPTM4B overexpression is a novel independent prognostic marker for metastatic ovarian tumors. International journal of gynecological cancer : official journal of the International Gynecological Cancer Society 22 (1):54-62. doi:10.1097/IGC.0b013e318234f9ac

Zhang G, Liang Y, Huang Y, Chen Y, Zhou R (2012) Elevated Lysosome-Associated Protein Transmembrane-4 -35 is an Independent Prognostic Marker in Pancreatic Carcinoma. Journal of International Medical Research 40 (4):1275-1283. doi:10.1177/147323001204000406

Zhang H, Wei Q, Liu R, Qi S, Liang P, Qi C, Wang A, Sheng B, Li L, Xu Y (2014) Overexpression of LAPTM4B-35: a novel marker of poor prognosis of prostate cancer. PloS one 9 (3):e91069. doi:10.1371/journal.pone.0091069.t001

Zhou L, He XD, Chen J, Cui QC, Qu Q, Rui JA, Zhao YP (2007) Overexpression of LAPTM4B-35 closely correlated with clinicopathological features and post-resectional survival of gallbladder carcinoma. European journal of cancer 43 (4):809-815. doi:10.1016/j.ejca.2006.10.025

Zhou L, He XD, Cui QC, Zhou WX, Qu Q, Zhou RL, Rui JA, Yu JC (2008) Expression of LAPTM4B-35: a novel marker of progression, invasiveness and poor prognosis of extrahepatic cholangiocarcinoma. Cancer letters 264 (2):209-217. doi:10.1016/j.canlet.2008.01.025
