# Supplementary material for: The relationship between LAPTM4B polymorphisms and cancer risk in Chinese Han population: a meta-analysis
Source: Springerplus. 2015 Apr 15;4:179. doi: 10.1186/s40064-015-0941-7 (PMC4408309; doi:10.1186/s40064-015-0941-7)
Supplement: Additional file 4: Table S4. — The stratified analysis for the estimation of association between LAPTM4B expression and prognosis in different type of cancer. [file 40064_2015_941_MOESM4_ESM.docx]

Table S4 The stratified analysis for the estimation of association between LAPTM4B expression and prognosis in different type of cancer

| Type of tumor | No. of studies | No. of population | HR | 95%CI | p | P(H) |
| --- | --- | --- | --- | --- | --- | --- |
| OS | | | | | | |
| Gynecological tumor | 4 | 413 | 3.846 | 2.482-5.96 | <0.001 | 0.036 |
| Digestive system | 4 | 370 | 3.965 | 2.543-6.183 | <0.001 | 0.107 |
| Others* | 2 | 380 | 1.703 | 1.136-2.553 | 0.010 | 0.167 |
| DFS | | | | | | |
| Digestive system | 3 | 272 | 3.615 | 2.197-5.949 | <0.001 | 0.043 |
| Others** | 2 | 307 | 3.716 | 1.698-8.133 | 0.001 | 0.751 |

*Others: breast cancer and NSCLC,**others: breast cancer and cerical carcinoma, OS: overall survival, DFS: disease-free survival, P(H):p-value for the test of heterogeneity
